# Colon importance in short bowel syndrome

**DOI:** 10.18632/aging.102447

**Published:** 2019-11-15

**Authors:** Cecile Lambe, Olivier Goulet, Lorenzo Norsa

**Affiliations:** 1 Pediatric Gastroenterology Hepatology and Nutrition Necker Enfants Malades Hospital, Paris, France; 2 Pediatric Hepatology Gastroenterology and Transplantation, ASST Papa Giovanni XXIII, Bergamo, Italy

**Keywords:** short bowel syndrome, intestinal failure, colon, parenteral nutrition

Short bowel syndrome is a rare condition that is either acquired late in life due to mesenteric ischemia, Crohn’s disease and cancer - or very early in life in infants with congenital malformations and necrotizing enterocolitis. As it is generally accepted that one can live without a colon with no major side effects, it is also generally accepted that what matters the most in short bowel syndrome is the remaining small bowel length and not so much the large bowel. As a matter of fact, a surgeon will work more towards preserving the small intestine than he will work towards saving the colon. After bowel resection, a physiological intestinal adaptation occurs that involves the small intestine and the colon. A few studies in adults have shown that the colon becomes an energy absorptive organ [1]. Our study published in 2019 was the first to demonstrate that the same adaptation process occurred in children [2]. We showed that to obtain the same absorption rate (70% of ingested calories), four times less small bowel length was needed in case of the presence of the whole colon compared to no colon at all. Conversely, for the same small bowel length, the predicted average absorption rate was twice more in the children with a preserved colon compared to the children without a colon. This has important clinical implications making possible – or not – the weaning of parenteral nutrition and the leading of a normal life. Now that the role of the colon as an energy absorptive organ is established, it is critical to better understand what mechanisms take place to reach this shift of function in order to contribute to improved management of these patients.

Several concepts have been developed through the years but it is only with the advent of the microbiota as a key-marker that we were able to better understand the adaptative mechanisms. The microbiota is a key element for the colon to become an “energy absorptive” organ. Firstly, through the process of malabsorbed carbohydrates fermentation: some bacteria are able to ferment carbohydrates and convert them into short-chain fatty acids such as butyrate, propionate and acetate. These short-chain fatty acids are released into the colon lumen by the bacteria and are then easily absorbed across the colonic mucosa by passive transfer into the blood circulation becoming an energy source [3]. But they also play a role in the stimulation of the colonic mucosa and in the release of growth factors such as GLP-2 [4]. The second mechanism is the capacity of the colon to absorb medium-chain triglycerides which are water-soluble contrary to long-chain fatty acids [5]. Thirdly, the colon – particularly the caecum – is responsible for the production of GLP-2 by the neuro-endocrine L-cells after stimulation by undigested nutrients. The GLP-2 plays an important role in the physiological intestinal adaptation in stimulating enterocytes growth, delaying gastric emptying and increasing the intestinal blood flow. Dipeptidyl peptidase degradation resistant GLP-2 analogues are now used in clinical practice in patients with short bowel syndrome and are more efficient in patients without a colon [6]. Also, the on-going discovery of the intestinal microbiota has revealed that some bacteria were able to produce dipeptidyl peptidase inhibitors (the same that are used in clinical practice in diabetes mellitus) which prevent the degradation of GLP-2 and GLP-1 by the dipeptidyl peptidase 4. It then becomes obvious that the colon as a host and organ - and the microbiota as an organ with a new function - are working together to preserve the individual eco-system and its self-maintaining capacity as a host. Whether this cooperation is enough depends on several other factors such as the ability of the individual in feeding oneself to provide energy to the bacteria, of the physicians to avoid prescribing antibiotics that will destroy the microbiota balance, and of the microbiota itself that can exceed its beneficial function by producing toxic substances leading to complications such as D-lactic acidosis.

All this stresses the necessity for conservative surgery of the colon at the time of the initial resection and for the restoration of an intestinal continuity as soon as possible when the colon has been saved. And it seems more and more mandatory to respect the intestinal microbiota by avoiding as much as possible the use of antibiotics in this setting.

Furthermore, the decryption of the intestinal microbiota – alike the decryption of the human genome - will help us to understand thoroughly and to respect those extraordinary adaptative systems that are crucial for any individual at every stage of his life.
